# Human Respiratory Syncytial Virus Infections among Hospitalized Children in Poland during 2010–2020: Study Based on the National Hospital Registry

**DOI:** 10.3390/jcm11216451

**Published:** 2022-10-31

**Authors:** Michał Rząd, Krzysztof Kanecki, Katarzyna Lewtak, Piotr Tyszko, Martyna Szwejkowska, Paweł Goryński, Aneta Nitsch-Osuch

**Affiliations:** 1 Department of Social Medicine and Public Health, Medical University of Warsaw, 3 Oczki Street, 02-007 Warsaw, Poland; 2 Institute of Rural Health in Lublin, 2 Jaczewskiego Street, 20-090 Lublin, Poland; 3 Department of Pediatrics with Medical Assessment, Medical University of Warsaw, Pediatric Teaching Clinical Hospital, 63A Żwirki i Wigury Street, 02-091 Warsaw, Poland; 4 National Institute of Public Health NIH—National Research Institute, 24 Chocimska Street, 00-791 Warsaw, Poland

**Keywords:** respiratory syncytial virus, epidemiology, hospitalization, national register, public health

## Abstract

Background: Human respiratory syncytial virus (RSV) is responsible for infections mainly affecting the lower respiratory tract in infants and young children after the first exposure. The aim of the study is to show up-to-date information on RSV hospitalization cases in Poland in children aged < 5 years. Methods: A retrospective, population-based study was conducted using data from hospital discharge records of patients hospitalized from 2010–2020. Results: The study group consisted of 57,552 hospitalizations of RSV children. The mean and median ages were 232 (95% CI: 230–234) and 132 (IQR 63–271) days. The mean annual hospitalization rate for patients with RSV infection was estimated to be 267.5 per 100,000, and the highest was observed in children < 1 year (1132.1 per 100,000). The mean annual hospitalization rate was significantly higher in patients living in urban than rural regions (*p* < 0.001). A statistically significant increase in the number of hospitalizations was observed (*p* < 0.0001) during the analyzed period. The seasonal pattern was found with the highest rates of hospitalizations in the January–March period. Conclusions: The increasing RSV hospitalization rate requires further research and may be the basis for urgent healthcare measures. The results may be helpful in comparative analyses in the European and global context.

## 1. Introduction

Human respiratory syncytial virus (RSV) infections are common worldwide. RSV is an enveloped, non-segmented negative-strand RNA virus that belongs to the Paramyxoviridae family [1,2]. As a result of infection, an inflammatory immune response is triggered, which causes bronchiolitis with necrosis of the airway epithelial cells, leading to their obstruction (by clogging with mucus and cell debris). Adverse effects of this reaction also include ciliary dysfunction (impaired mucus clearance), swelling of the respiratory tract, and decreased lung compliance [2,3,4].

Two major subtypes of RSV can be distinguished: A and B. In one epidemic season, multiple genotypes are observed which can cause diseases of different severity. Individual strains of the RS virus differ from each other in terms of immunopathogenicity [5,6]. This suggests that the phenotype of the disease is composed of the patient’s response and the characteristics of the virus itself. The RSV RNA-dependent replication mechanism is highly error-prone. This causes quite frequent mutations which make it virulent. This results in potential antivirals or vaccines being ineffective [2].

Almost every child (over 95%) experiences RSV infection up to the age of 2 [2,5]. It is estimated that first-exposure lower respiratory tract infection (LRTI) occurs in 15–50% of infants and young children, and approximately 3% may require hospitalization for this reason. Infants aged 2 to 6 months are most at risk, although in some regions the highest incidence of LRTI is seen in patients aged 6–11 months and even those of 1 to 2 years of age. In 5–10% of hospitalized pediatric patients, the disease may require a stay in the intensive care unit (ICU) [4,7]. Among children hospitalized for RSV infection, the most common complications are acute otitis media (48%), pneumonia (33%), and conjunctivitis (11%) [8]. Patients with RSV in childhood are at a greater risk of suffering from asthma when older [1,9].

It is estimated that 33.1 million RSV infections occurred worldwide in 2015. They caused approximately 3.2 million hospital admissions and 59,600 in-hospital deaths in children aged < 5. Among the aforementioned group, there were 1.4 million hospital admissions and 27,300 deaths among children below 6 months [10]. Using data from 32 countries, the meta-analysis estimated the rate of hospitalization due to acute RSV respiratory infection at 4.37 (95% CI: 2.98–6.42) per 1000 children < 5 years, including 19.19 (95% CI: 15.04–24.48) per 1000 children < 1 year. Global mortality estimates for this infection were 6.21 (95% CI: 2.64–13.73) per 1000 children < 5 years and 6.60 (95% CI: 1.85–16.93) per 1000 children < 1 year [11].

Healthy infants and children rarely die in developed countries. Mortality rates are significantly higher in patients in developing countries and in those with underlying medical conditions. At least 66,000 children under the age of 5 die worldwide. Most serious cases and deaths occur in children under the age of two [5,6,7,12]. Indoor congestion promotes RSV infection. Most often this is when temperatures are the lowest and rainfall is the highest in the year. This results in an increase in the transmission of the virus [2]. Therefore, in temperate climates, the annual outbreaks of epidemics occur in late autumn, winter, or early spring (in the northern hemisphere between October and May), while in cold climates they occur all year round [5,6]. In tropical regions, the time of RSV outbreaks in a year is different, therefore it is difficult to predict the seasonality of the virus in these zones [2].

Data on the epidemiology of RSV infections in Poland are considerably limited. Depending on the hospital’s level of reference, the number of hospitalizations of children in Poland due to pneumonia varies between 9.5% and 26.6%, while for bronchitis it ranges from 9.3% to as much as 64.3%. Between 2012 and 2017, a nearly threefold increase in the frequency of hospitalizations due to pneumonia was observed in the hospital with the highest reference level, which indicates significant public health implications of this issue [13]. Based on 802 samples from Polish patients aged 0–14 years, it was found that influenza viruses accounted for 48.0% and influenza-like viruses for 52.0% of all positive samples. Among influenza-like viruses, the predominant etiological factor was RSV, which accounted for as many as 96.2% of samples [14].

International guidelines of scientific societies and virology experts recommend controlling the epidemiology of RSV infections at a national level and then (based on the data collected) at an international level [15]. Due to the importance of the analyzed phenomenon in the context of public health, financial and health costs for the entire healthcare system, and the lack of accurate data to date, there is a need for a more detailed analysis of the epidemiology of RSV infections among children in Poland.

The study was conducted in order to present current information on RSV-related hospitalizations in Poland in a group of patients below 5 years of age.

## 2. Materials and Methods

### 2.1. Patients and Methods

This study, which is a retrospective, population-based study, was performed on the basis of hospital discharge records of patients < 5 years of age, who had been diagnosed with an RSV infection. In total, 57,552 records of patients with RSV were analyzed in the study. The data were collected from 2010 to 2020 in a Nationwide General Hospital Morbidity Study carried out by the National Institute of Public Health NIH-National Research Institute in Poland. The study focused on RSV hospitalization cases with primary and secondary RSV infection diagnoses. All hospitals in Poland, except for psychiatric facilities, are obliged to share hospital discharge data with the Institute. Data are anonymous and include sex, date of birth, place of residence, data on hospital admission and discharge, and the International Classification of Diseases (ICD-10) code.

Despite being anonymous, the data allowed for the analysis of hospitalization frequency in individual patients. It was assumed that the hospital diagnosis of RSV infection was made according to the most current and commonly used criteria, even though the database did not provide direct information on the laboratory confirmation of RSV. The inclusion criteria were ICD-10 codes: ‘J12.1—respiratory syncytial virus pneumonia’, ‘J20.5—acute bronchitis due to respiratory syncytial virus’, ‘J21.0—acute bronchiolitis due to respiratory syncytial virus’, or ‘B97.4—respiratory syncytial virus as the cause of diseases classified elsewhere’.

The local Bioethics Committee was informed about the study; however, its approval was not required due to the retrospective and noninvasive design of the research.

### 2.2. Statistical Analysis

Statistical analyses were performed with Statistica (TIBCO Software Inc, Palo Alto, CA, USA) [16] and WINPEPI [17]. We computed means, medians, and ranges for continuous variables, and counts and percentages for categorical variables. Means and 95% confidence intervals or medians and interquartile ranges were calculated for continuous variables with normal or non-normal distribution, respectively. We analyzed counts and percentages for nominal variables. The rates of RSV infection hospitalizations were expressed as the estimated number of hospitalizations in patients below 5 years of age per 100,000 per year. Data taken from the Central Statistical Office of Poland [18] were used to calculate these rates. We used linear regression to assess the trends and Student’s *t*-test to analyze the assumption that, in a public health study, there is normal distribution in samples that are sufficiently large [19]. In cases when the normality assumptions could not be observed, non-parametric tests were used. It was assumed that the two-sided *p*-value < 0.05 was statistically significant.

## 3. Results

In total, 57,552 records of RSV hospitalizations were analyzed in this study. In the analyzed period, 92.6% of patients were hospitalized with RSV infection only once. The study group consisted of 32,897 males (57.2% of all patients) and 24,649 females (42.8% of all patients), and in 6 cases, sex was not specified as male or female.

The ICD-10 code of ‘J12.1—respiratory syncytial virus pneumonia’ was reported in 53.2% of the analyzed cases, ‘J21.0—acute bronchiolitis due to respiratory syncytial virus’ was reported in 32.6% of cases, ‘J20.5—acute bronchitis due to respiratory syncytial virus’ was reported in 18.3% of cases, and ‘B97.4—respiratory syncytial virus as the cause of diseases classified elsewhere’ was reported in 1.2% of cases.

A significant predominance of male patients was observed in the study group when compared to the general population (*p* < 0.0001).

**Figure 1:**
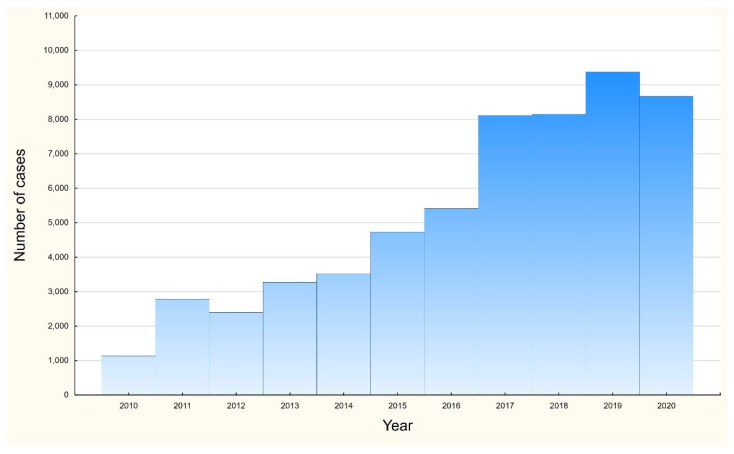
Number of hospitalizations among RSV children below 5 years in Poland, 2010–2020.

In the study group, the mean and median ages were 232 (95% CI: 230–234) and 132 (IQR 63–271) days. There were no significant differences in mean age in the study group; however, in the group of children below one year of age, significant differences in mean age were observed between males and females (128.5 vs. 119.5; *p* < 0.0001). A statistically significant increase in the number of hospitalizations was observed in the study during the analyzed period, as presented in Figure 1; adjusted R^2^ = 0.93, *p* < 0.0001.

**Figure 2:**
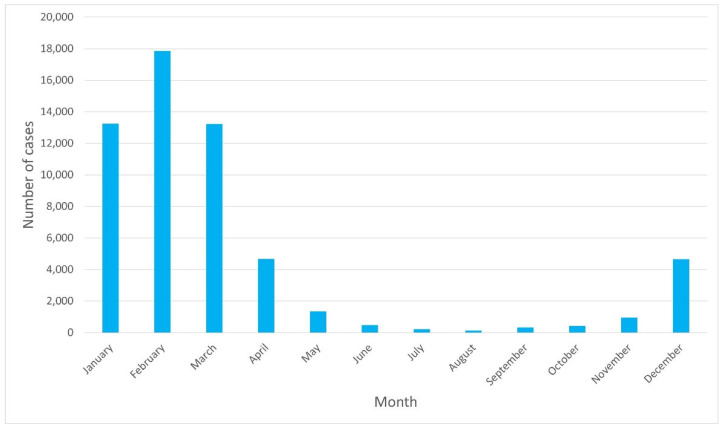
Number of RSV hospitalization cases per month.

The highest hospitalization rates were observed from January to March, and the lowest ones were in August. Thus, a seasonal pattern can be assumed, as presented in Figure 2.

**Figure 3:**
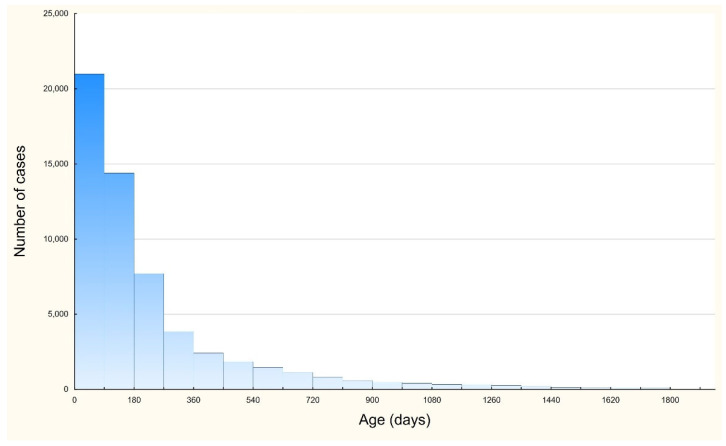
Number of RSV infection hospitalization cases per children age.

Figure 3 shows that the majority of cases were observed in patients aged <1 year. In this age group, we observed 47,041 cases (81.7% of all cases). In the subgroup of children under 1 year of age, children aged 0–90 days represented 36.8% of this group, and those aged 0–180 days accounted for 61.7% of the group.

The mean rate of hospitalizations in the group of patients with RSV infections was established at 267.5 per 100,000 annually. The mean annual hospitalization rate was significantly higher in patients from urban regions than in those from rural regions (267 per 100,000 vs. 256 per 100,000; *p* < 0.001). Place of living was unknown in 1146 hospitalization cases.

**Figure 4:**
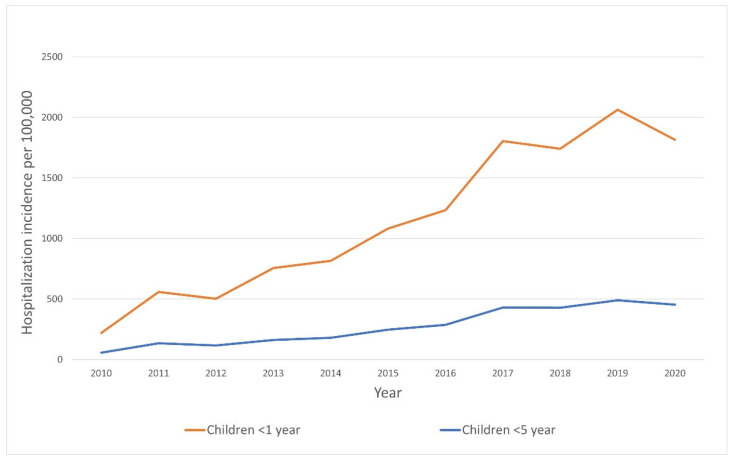
Hospitalization rate trends due to RSV infection in Poland, 2010–2020.

In children under 1 year of age, the mean hospitalization rate for patients with RSV infection was estimated to be 1132 per 100,000 per year. There was no significant difference in mean annual hospitalization rate between patients from urban and rural regions. A significant increase in the number of hospitalizations in this subgroup (under 1 year of age) was observed in the analyzed period (adjusted R^2^ = 0.90, *p* < 0.0001). Changes in hospitalization rates over the analyzed period are presented in Figure 4.

In the analyzed period, 45 deaths were recorded (0.08% of all patients): 22 males, and 23 females. The mean and median ages in this subgroup were 319 days (95% CI: 218–421) and 162 days (IQR: 99–482). 26 deaths were reported in urban regions and 18 deaths in rural regions, in one case place of living was unknown.

## 4. Discussion

The respiratory syncytial virus is one of the most frequent reasons of acute lower respiratory tract infections in children worldwide so it is important to obtain epidemiological RSV data as precisely as possible. Based on the national hospital disease registers, the number of hospitalizations in Poland in 2010–2020 due to RSV infection was 57,552. In our study, the mean annual hospitalization rate for patients with RSV infections was estimated to be 267.5 per 100,000, and in children under 1 year of age, the rate was estimated to be 1132 per 100,000. The above number of patients may be significant. However, the estimated incidence rate of hospitalizations due to RSV infection among children in Poland appears to be low compared to the data from the scientific literature. In a study based on data from six European countries, the incidence of hospitalizations in 0–2 months old children was above 4000 per 100,000, while in the group of 1–2 year-olds, it ranged from 130 to 1050 per 100,000 [20]. In an article aggregating data from 32 countries, the estimated incidence of hospitalization in 2015 in children < 5 years of age was 437 per 100,000 children, and among children < 1 year, even 1919 per 100,000 children [11]. The discrepancy between the data we present and those revealed in studies from other countries may probably be due to the limited number of tests and certain verification of RSV infection among children in Poland.

At the same time, a significant increase (as shown in Figure 1) in the number of identified cases was observed over the period analyzed, both among the entire study group and among infants. This phenomenon may be due to a number of factors, such as, among others, the increasing availability of rapid diagnostic tests characterized by a sensitivity of 75.3% and a specificity of 98.7% (especially in the era of the COVID-19 pandemic and the need to efficiently distinguish the etiological causes of viral infections among children), improved diagnostic performance, changes in virus virulence and other unidentified factors [21].

In the present study, children aged 0–90 days accounted for approximately one-third of all hospitalized patients. It may be similar to other studies where the median age of RSV was 8.4 weeks and the highest hospitalization rate was among children aged 0–2 months [20,22].

The mean hospitalization rate was significantly higher in patients from urban regions than in those from rural regions. There may exist a relationship between RSV incidence and the place of residence. Socioeconomic status or other factors related to the availability of RSV tests are likely to cause the disease to go undiagnosed in some cases. Work on this issue may significantly contribute to the improvement of the health condition of the rural population in Poland. On the other hand, living in an urban setting, with potentially more polluted air, may favor the development of respiratory diseases more easily.

The current study reports the predominance of male patients (57.2% of all patients). The predominance of males in relation to females was similarly reported in a recent study from the Middle East and North Africa [23]. A study from Iran likewise reported that male infants with RSV are more predominant [24]. In a cohort study from the USA, the number of female patients was 40.5% of RSV cases [25]. The predominance of the male sex (OR 1.23) in infections caused by RSV is also confirmed by numerous meta-analyses, distinguishing this fact as a risk factor for the disease. The other risk factors include: prematurity (OR 1.96), low birth weight (OR 1.91), having siblings (OR 1.60), maternal smoking (OR 1.36), history of atopy (OR 1.47), no breastfeeding (OR 2.24) and crowding (OR 1.94) [26]. The latest meta-analysis indicates the following among the most important factors: age, male gender, winter season, and environmental factors such as cold temperatures, higher relative humidity, high concentrations of benzene, exposure to tobacco, and living in urban areas [27].

Globally, in 2015, 59,600 hospital deaths associated with RSV infection were reported in children < 5 years of age [10]. In another study from the USA, based on national mortality data sets from the years 1999–2018, a mean of over 6500 underlying respiratory deaths per year was associated with RSV, including 96 deaths annually among children below 1 year of age [28]. In the current study, 45 deaths were reported (0.08 % of all patients). However, this estimate is associated with considerable uncertainty. In our study, we attributed all mortality rates to the RSV during the RSV season. However, the actual overlap between RSV, influenza, and other respiratory pathogens may be greater than in our limited data. We did not take into account the possible seasonal circulation of various respiratory pathogens. This may have resulted in an overestimation of the overall RSV mortality. In another study with 114 patients aged 0–59 months with symptoms of respiratory tract infection, co-infections were detected in nine (8%) patients [29]. In our study, we also observed more deaths due to infection with RSV in urban areas (26 cases to 18 cases in rural areas). This is likely due to unknown territorial factors, although differences in access to healthcare should also be considered.

The available data show that 92.6% of patients were admitted to the hospital only once in the analyzed period. A growing number of hospital admissions due to RSV infections indicates a very high burden on the hospital system in Poland, and it can lead to a presumption that the burden will increase further as access to and the use of health services increases with socio-economic development. Other studies show that the mean cost of an episode for inpatient and outpatient treatment without control was EUR 3452 and EUR 299, respectively, rising to EUR 8591 and EUR 2191, with follow-up for up to 2 years after the first event [30]. Thus, treatment of RSV infections is a considerable challenge for hospitals and public health, as it requires significant financial resources and season-oriented planning, with regard to human resources, adequate medicines, and pediatric care.

In our study, seasonality was found with the highest number of hospitalizations in January-March, and the lowest in August, as shown in Figure 2. In countries with a temperate climate (such as Poland), the season of infections due to RSV typically occurs in the winter months, from October/November until March/May, while the peak incidence is in January/February [2]. In the UK, the RSV season is defined as the period from October to March with a peak incidence in December [5,31,32]. In two studies from Switzerland and Croatia, the increased incidence occurred between November and March, with the peak incidence in January [33,34]. The results presented in our study confirm trends observed in other European countries.

This data can be particularly useful when undertaking public health measures in relation to prevention. For example, the implementation of prophylaxis with the monoclonal antibody—palivizumab. Prophylaxis with palivizumab was associated with a reduction in all-cause mortality and hospitalization for the respiratory syncytial virus in high-risk premature infants [35]. As another study shows, targeted prevention of RSV in infants at risk of hospitalization in the first year for RSV infection resulted in an increase in QALY by 0.02 (0.931 vs. 0.929) per patient at an additional cost of EUR 472 compared to no prophylaxis (ICER 214,748 EUR/QALY). The ICER drops below the threshold of EUR 80,000 per QALY when the cost of RSV prevention is lowered from EUR 928 (baseline) to EUR 406 per unit. At a cost of EUR 97 per unit, RSV prophylaxis would be a saving [36]. The presented data on the seasonality of RSV cases may be particularly useful in taking measures to reduce healthcare costs related to the treatment of this disease among the youngest patients.

Our study had several limitations. During the study, the correctness of diagnosis and disease reporting was not verified, which may distort the results obtained. In addition, the national database, which was the original source of data, did not include information on the virological confirmation of a particular case. Moreover, there has been no initial evaluation of ICD-10 coding practices. According to ICD-10 coding guidelines, RSV requires identification. Tests for RSV are available, and there are no restrictions on their use. However, some patients are not tested or they are tested too late to confirm the diagnosis, so they may be admitted to the hospital with other diagnoses. Furthermore, only hospital discharge records were included in the database, meaning that some of the outpatients with RSV were excluded from the study. On the other hand, the considerable length of the observation period and the large size of the data sample obtained from the National Register of Hospital Morbidity are of great benefit to this study.

## 5. Conclusions

To the best of the authors’ knowledge, this study presents, for the first time, the aspects of RSV based on the national hospital morbidity registry and presents the latest data on RSV epidemiology in Poland. Globally, RSV is a common cause of LRTI in children and the leading cause of hospitalization in young children, placing a heavy burden on healthcare services. Most hospital admissions and in-hospital deaths due to RSV-LTRI occur in children under the age of one year.

The data presented show a growing problem of RSV infections among children <5 years of age in Poland, which poses a serious challenge for public health, the healthcare system, and practicing physicians.

The results of this study may be helpful in comparative analyses in the European and global context and in taking actions aimed at improving the health condition of the Polish population.

## Data Availability

Nationwide General Hospital Morbidity Study, National Institute of Public Health NIH-National Research Institute, Warsaw, Poland.
